# Stellate ganglion block relieves acute lung injury induced by severe acute pancreatitis via the miR-155-5p/SOCS5/JAK2/STAT3 axis

**DOI:** 10.1186/s40001-022-00860-3

**Published:** 2022-11-04

**Authors:** Lei Wang, Na Yuan, Yuanli Li, Qinqin Ma, Ying Zhou, Zhifei Qiao, Shutie Li, Chunyan Liu, Liqian Zhang, Meng Yuan, Jianjing Sun

**Affiliations:** 1 Department of ICU, The First Affiliated Hospital of Hebei North University, Qiaoxi District, No.12 Changqing Road, Zhangjiakou, 075000 Hebei China; 2 Department of Radiotherapy, The First Affiliated Hospital of Hebei North University, Zhangjiakou, 075000 Hebei China; 3 Department of Emergency, The First Affiliated Hospital of Hebei North University, Zhangjiakou, 075000 Hebei China; 4 Department of Gastroenterology, The First Affiliated Hospital of Hebei North University, Zhangjiakou, 075000 Hebei China

**Keywords:** Stellate ganglion block, Acute lung injury, Severe acute pancreatitis, miR-155-5p, SOCS5

## Abstract

Acute lung injury (ALI), a prevalent complication of severe acute pancreatitis (SAP), is also a leading contributor to respiratory failure and even death of SAP patients. Here, we intended to investigate the function and mechanism of stellate ganglion block (SGB) in ameliorating SAP-induced ALI (SAP-ALI). We engineered an SAP-ALI model in rats and treated them with SGB. HE staining and the dry and wet method were implemented to evaluate pathological alterations in the tissues and pulmonary edema. The rats serum changes of the profiles of TNF-α, IL-6, IL-1β, and IL-10 were examined. The profiles of miR-155-5p and SOCS5/JAK2/STAT3 were detected. Functional assays were performed for confirming the role of miR-155-5p in modulating the SOCS5/JAK2/STAT3 pathway in pulmonary epithelial cells. Our findings revealed that SGB vigorously alleviated SAP rat lung tissue damage and lung edema and lessened the generation of pro-inflammatory cytokines TNF-α, IL-6, and IL-1β. SGB enhanced SOCS5 expression, hampered miR-155-5p, and suppressed JAK2/STAT3 pathway activation. As evidenced by mechanism studies, miR-155-5p targeted the 3′UTR of SOCS5 and repressed its expression, hence resulting in JAK2/STAT3 pathway activation. During animal trials, we discovered that SGB ameliorated SAP-ALI, boosted SOCS5 expression, and mitigated the levels of pro-inflammatory factors and miR-155-5p in the plasma. In vitro, miR-155-5p overexpression substantially facilitated pulmonary epithelial cell apoptosis, inflammation, and JAK2/STAT3 pathway activation and restrained SOCS5 expression. All in all, our work hinted that SGB could modulate the miR-155-5p/SOCS5/JAK2/STAT3 axis to alleviate SAP-ALI.

## Introduction

Pancreatin activated in the pancreas will trigger pancreatic autodigestion and thus result in acute pancreatitis (AP), which is usually correlated with cholelithiasis, binge-eating, and excessive drinking [1]. An aberrant hike in serum amylase is a typical indicator of AP. As per the severity, AP can be categorized into mild, moderate, and severe. Severe acute pancreatitis (SAP) features pancreatic necrosis, systemic inflammation, and even organ dysfunction [2]. Acute lung injury (ALI), a prevailing severe inflammatory reaction, is also an essential contributor to AP patients’ death. It is estimated that the mortality rate of AP elicited by ALI and acute respiratory distress syndrome (ARDS) can reach as high as 60% [3]. In the teeth of scanty efficacious intervention strategies for SAP-ALI with very sophisticated pathogenesis, we conducted the research in the hope that novel therapies could be uncovered to ameliorate the existing poor prognosis of SAP-ALI.

The stellate ganglion comprises the sixth and seventh cervical vertebrae as well as the first thoracic sympathetic ganglion, functionally belonging to the sympathetic nerve. When stimulated by inflammation and serious pressure, the body will experience imbalances between the sympathetic nerve and the parasympathetic nerve, which then elicits diseases concerning sympathetic nerve regulation like arrhythmia [4, 5]. SGB blocks the sympathetic nerve and redresses the vegetative nerve imbalance through the injection of anesthetic drugs into stellate ganglion tissues, thus meeting its goal of stabilizing the internal environment of the body [6]. SGB, a novel therapy with great potential, has been demonstrated to greatly enhance the spatial learning and memory capabilities of rats stimulated by unpredictable chronic stress and alleviate their depression-like behaviors [7]. SGB vigorously hampers inflammatory factor release and mitigates the clinical symptoms of patients suffering from ulcerative colitis (UC) [8]. Concentrating on SGB, we probed whether it could be applied in SAP-ALI treatment in the research.

microRNAs (miRNAs), endogenous non-coding small molecule RNAs, modulate multiple biological functions like cell proliferation, apoptosis, differentiation, angiogenesis, inflammation, and infection [9]. As demonstrated by many scholars, miRNAs are the underlying targets for AP treatment. miR-92b, miR-10a, and miR-7, which are down-regulated in AP patients, can be utilized for early AP diagnosis. miR-551b-5p, down-regulated in AP patients with complications or a low level of plasma calcium, enables us to differentiate the degrees of the disease [10]. miR-214-3p, whose expression is uplifted in the pancreas of hyperlipidemia pancreatitis (HP) rats, dampens PTEN and upregulates Akt to step up HP-elicited pathological alterations and inflammation [11]. miR-155-5p, another member of the miRNA family, displays a pro-inflammatory function in a few inflammatory diseases like hippocampal inflammation in acute seizures [12], Parkinson’s disease [13], and lipopolysaccharide-induced acute lung injury [14].

Suppressors of cytokine signaling (SOCS) can suppress their downstream signaling to regulate the signaling speed and time of various cytokine receptors [15]. For instance, SOCS5, a pivotal member of the SOCS family, boasts a central SH2 domain and a C-terminal conserved domain and has a special kinase-inhibitory region (KIR) domain, which can impede kinase activity [16]. miR-151a-3p overexpression hinders SOCS5 and initiates the JAK2/STAT3 pathway to abate MC3T3-E1 cell viability and spur postmenopausal osteoporosis progression [17]. The Janus kinase 2/signal transducer and activator of transcription 3 (JAK2/STAT3) pathway mediates proliferation, immunity, programmed cell death and other processes [18]. Interestingly, hydrostatin-SN10 cramps the IL-6-triggered activation of the JAK2/STAT3 pathway, lessens cell apoptosis, inflammation, and oxidative stress and mitigates lung damage induced by pancreatitis [19]. Therefore, a probe into the JAK2/STAT3 pathway mediated by SOCS5 helps us better understand the exact mechanism of lung damage elicited by pancreatitis.

In a nutshell, the outstanding an-inflammatory function of SGB has been verified. Here, we went further into the therapeutic function of SGB in SAP-ALI and discovered that SGB considerably ameliorated ALI in the SAP SD rat model, suppressed miR-155-5p expression and JAK2/STAT3 pathway activation, and bolstered SOCS5 expression. Thus, we conjectured that SGB could modulate the SOCS5-JAK2/STAT3 regulatory axis mediated by miR-155-5p to alleviate SAP-ALI.

## Materials and methods

### Cell culture and transfection

MLE-12 cells, lung epithelial cells derived from mice, were ordered from the American Type Culture Collection (ATCC, Rockville, MD, USA). Inoculated into an RPMI1640 medium incorporating 10% inactivated fetal bovine serum (FBS, HyClone, Logan, UT, USA), 1 × 10^5^/mL cells were cultured with 5% CO_2_ at 37 ℃ and passed every two or three days. Cells in the logarithmic growth phase were digested, passed, and seeded into 12-well plates with a density of 5 × 10^6^/well. As the cells met 80–90% confluence, miR-155-5p mimics, SOCS5 overexpression plasmid, and their corresponding negative controls were transfected into them as per the instructions of Lipofectamine^®^3000 (Invitrogen; ThermoFisher Scientific, Inc.). The cells were grown in an incubator with 5% CO_2_ at 37 ℃. LPS (1 μg/mL; Sigma-Aldrich, St. Louis, MO, USA) was administered to treat the MLE-12 cells for the construction of an in vitro pulmonary epithelial cell damage model.

### CCK8 assay

MLE-12 cells, inoculated into 96-well plates with a density of 5 × 10^3^/well, were dealt with LPS (1 μg/mL) for 24 h. Then, 10 μL of CCK8 reagent (Beyotime, Shanghai, China) was given for 2 h incubation at 37 ℃. A microplate reader was exploited to gauge the absorbance at 450 nm.

### Animal model establishment

Thirty SD rats 220–250 g in weight, ordered from the Animal Experimental Center of Zhangjiakou Medical College (Zhangjiakou, China), were reared in an environment of 50–60% humidity at 25 ℃ for seven days under a 12 h light/dark cycle, given sufficient food and water in Laboratory animal management department of Zhangjiakou Medical College. The SGB model: eight hours after the SD rats were put into a fast, they were intraperitoneally injected with pentobarbital sodium (40 mg/kg) for anesthesia; they were then fixed on the operating table in a supine position; with a 1 cm incision made in the middle of their necks, the subcutaneous tissues, fascia, and muscle were isolated; when the right common carotid artery was seen, the right sympathetic nerve trunk was isolated and exposed under a microscope; the sympathetic nerve trunk of the broken neck was treated 3 mm below the superior cervical ganglion, the severed end was ligated, and the incision was sewed up. The rats manifested a drooping right eyelid, enophthalmos, narrowed pupils and other Horner symptoms after they woke up, which verified the success in the establishment of the SGB model. The AP model: 12 h after the fast, the rats were intraperitoneally transfused with pentobarbital sodium (40 mg/kg) for anesthesia; a cut was made right in the middle of the abdomen, and sodium taurocholate (3.5%, 1 ml/kg, Sigma, St. Louis, MO, United States) was retrograde-injected into the biliary pancreatic duct to elicit AP in the animals. As the rats displayed symptoms like drinking a lot of water, poor diet, lusterless and tangled hair, short breath, and lung noises, we confirmed the success. Twenty-four hours later, the rats were narcotized through the intraperitoneal injection of pentobarbital sodium (100 mg/kg) and euthanized. We harvested their abdominal aortic blood and lung tissues for the following analysis.

### Hematoxylin–eosin staining (HE)

The harvested lung tissues were immobilized employing 4% paraformaldehyde at 4 ℃ for 24 h, embedded in paraffin, and sectioned in the thickness of 5 μm. Then, the slices were dewaxed, dehydrated with gradient alcohol, and dyed with hematoxylin and eosin solution (HE). A microscope (200 × magnification, Olympus Corporation, Tokyo, Japan) was deployed to monitor pathological alterations in the lung tissues. In accordance with the Hofbauer scoring system, we observed pathological changes like edema, hemorrhage, and inflammatory infiltration in the tissues. Zero indicates normality. One point suggests that the above alterations appear in 25% of the visual field. Two points mean the changes covering 50% of the field. Three points indicate the changes covering 75% of the field. Four points denote that those alterations are seen in the whole field.

### Wet-to-dry (W/D) ratio

The right pulmonary tissues of the rats were flushed in PBS and dried, with the wet weight (W) gauged. Then, the tissues were heated in a constant temperature oven at 70 ℃ for 72 h, with the dry weight (D) measured. The level of lung edema was examined with this formula: pulmonary edema index = W/D.

### Arterial blood gas analysis

Uncoagulated rat abdominal aortic blood samples incorporating heparin were put into the blood gas analyzer (Beckman Coulter, Inc., USA) to examine the arterial partial pressure of oxygen (PaO_2_), the partial pressure of carbon dioxide (PaCO_2_), and the PH value.

### Serum amylase detection

Serum amylase is a typical indicator of acute pancreatitis diagnosis. Biocompare Company (CA, USA) supplied us with the amylase ELISA kit, which was adopted to examine serum amylase in the rats.

### BALF collection and inflammatory cell counting

The bronchoalveolar lavage fluid (BALF) of the rats was obtained for quantifying inflammatory cells. After the rats were narcotized, as mentioned in 2.1, we put the front end of a bronchoscope into the opening at the lateral segmental bronchus in the middle lobe of the right lung, injected 2 mL of 37 ℃ normal saline, and conducted lavage three times. PMNs, extracted out of BALF, were purified and centrifuged at 1000 r/min for 10 min. With the supernatant harvested, RMPI-1640 was administered to resuspend the cells. A hematocyte counter (Beckman Coulter, Inc) was utilized to count inflammatory cells in BALF. Cytospin (Thermo Fisher Scientific, Waltham, USA) was harnessed to centrifuge 100 μl of BALF onto slides. After getting dried, the slides were dyed employing the Protocol HEMA-3 Cell Staining Kit (Fisher, Pittsburg, PA).

### Enzyme linked immunosorbent assay (ELISA)

The rat lung tissues were lysed employing a lysis buffer supplemented with the protease inhibitor and centrifuged at 2500 r/min and 4 ℃ for 20 min. With the supernatant obtained, the ELISA kits (R&D Systems, Minneapolis, MN, USA) were utilized to determine the levels of inflammatory cytokines (TNF-α, IL-6, IL-1β, IL-10). We harvested the plasma and operated the ALT and AST kits (Nanjing Jiancheng Institute of Biotechnology, Nanjing, China) to gauge ALT and AST expression levels in the samples. A Power Wave microplate reader (Bio-TEK, USA) was exploited to examine the OD value at 450 nm.

### Quantitative reverse transcription PCR (qRT-PCR)

Total RNA was extracted out of the cells with the use of TRIzol reagent (Invitrogen, Carlsbad, CA, USA), with the RNA purity determined. Then, the RevertAid First Strand cDNA Synthesis Kit (Thermo Fisher Scientific, Waltham, MA, USA) was adopted to reverse-transcribe the mRNA into cDNA, and the miScript II Reverse Transcription Kit (Qiagen, Hilden, Germany) was employed to reverse-transcribe the miRNA into cDNA. qRT-PCR was implemented with the assistance of SYBR Green PCR Master Mix (Roche) or the miScript SYBR^®^ Green PCR Kit (QIAGEN, Dusseldorf, Germany) in the ABI Step-One PlusTM Real-Time PCR System (Applied Biosystems). GAPDH was taken as the internal parameter of SOCS5. The primer sequences are detailed in Table 1. The relative profile of each gene was calculated through the 2^−ΔΔCT^ method.

**Table 1:** Primer sequences

Name	Primer sequences
miR-155-5p	Forward:5′-UAAUACCGUCUUAAAACCGU-3′
	Reverse:5′-AGAGAGAGAGGTCCTGAGGG-3′
SOCS5	Forward:5′- TCAATGGATAAAGTGGGGAAAATGTGG-3′
	Reverse:5′- TACTTTGCCTTGACTGGCTCTCGTTCTA-3′
GAPDH	Forward:5′-TGGTTGAGCACAGGGTACTT-3'
U6	Reverse:5′-CCAAGGAGTAAGACCCCTGG-3'
	Forward:5′ CAGCACATATACTAAAATTGGAACG-3’
	Reverse:5′- ACGAATTTGCGTGTCATCC-3’

### Dual luciferase activity assay

The biological information website Targetscan (http://www.targetscan.org/vert_72/) discovered that SOCS5 was an underlying target of miR-155-5p. MLE-12 cells, inoculated into 24-well plates with a density of 5 × 10^5^/well, were transfected along with SOCS5-WT, SOCS5-MT, miR-155-5p mimics, and their respective negative controls using Liposome 2000 reagent (Invitrogen; Thermo Fisher Scientific, Inc. Waltham, MA, USA). The luciferase activity was gauged as instructed by the manufacturer (Promega, Madison, WI, USA). The experiment was duplicated three times.

### Western blot

The rat lung tissues were subjected to homogenization through a homogenizer containing a reagent for cell nucleus and cytoplasm protein extraction (Sigma-Aldrich; Merck KGaA). We harvested MLE-12 cells. RIPA (Beyotime, Shanghai, China) was administered to the tissue homogenate and cells, and the total protein was separated. Bicinchoninic acid assay (Pierce, Rockford, USA) was carried out for protein quantification. Protein samples (50 µg) were isolated through 12% SDS-PAGE and then moved onto polyvinylidene fluoride (PVDF) membranes (EMD Millipore, Billerica, MA, USA). After being sealed with 5% skimmed milk for an hour at room temperature (RT), the membranes were incubated along with primary antibodies Anti-SOCS5 antibody (1:1000, sc-100858, Santa Cruz Biotechnology, USA), Anti-JAK2 (phospho Y1007) antibody (1:1000, Ab195055, abcam, USA), Anti-JAK2 antibody (1:1000, ab108596, abcam, USA), Anti-STAT3 (phospho Y705) antibody (1:1000, ab76315, Abcam, USA), Anti-STAT3 antibody (1:1000, Ab68153, Abcam, USA), Anti-Bcl2 antibody (1:1000, Ab194583, Abcam, USA), Anti-Bax antibody (1:1000, Ab32503, Abcam, USA), Anti-Caspase3 antibody (1:1000, Ab184787, Abcam, USA), and Anti-GAPDH antibody (1:1000, ab181602, Abcam, USA) overnight at 4 ℃. TBST was taken to flush the membranes twice, which were later incubated along with the fluorescein-labeled goat anti-rabbit IgG secondary antibody (1:3000, ab150077, abcam, USA) at RT for an hour and rinsed another three times. The protein bands were examined employing the ECL Western blot kit (Amersham Biosciences, UK), and the Image Lab v5.2.1 software (Informer Technologies, Inc.) was introduced for quantification.

### Statistical analysis

The GraphPad Prism 8 software (GraphPad Software, Inc., city, state) was introduced to analyze statistical differences, with the measurement statistics presented as mean ± standard deviation (X ± S). One-way ANOVA was taken for comparison among multiple groups, while Tukey post hoc test was implemented to compare two groups. *P* < *0.05* was regarded as statistically meaningful.

## Results

### SGB could efficaciously alleviate SAP-ALI

**Fig. 1:**
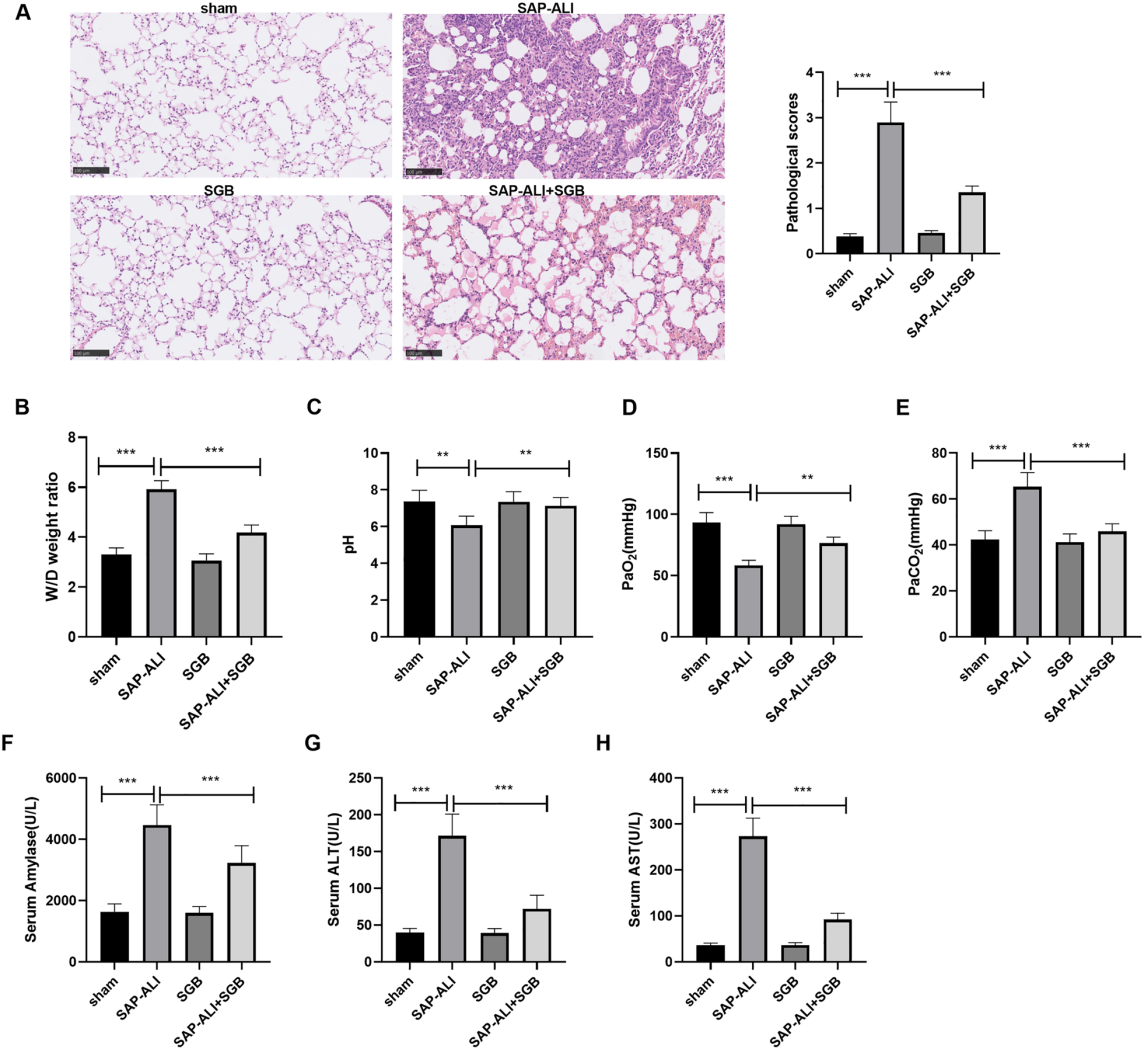
SGB could efficaciously ameliorate SAP-ALI. An SAP rat model was engineered through sodium taurocholate, and an SGB rat model was set up via cervical sympathetic nerve trunk dissection. The rats’ lung tissues and abdominal aortic blood were harvested. **A** HE staining monitored pathological alterations in the rat lung tissues. **B** The dry and wet method evaluated lung edema in the rats. **C**–**E** Arterial blood gas analysis determined the levels of PH, PaO_2_, and PaCO_2_ and assessed the ventilation function in the rats. **F**–**H** ELISA confirmed the levels of amylase, ALT, and AST in the rat serum. **P < 0.01, ***P < *0.001* (vs. the sham group), **P < 0.01, ***P < *0.001* (vs. the SAP-ALI group). *N* = 5

To confirm whether SGB could ameliorate SAP-ALI, we engineered an SAP model in rats and treated the animals with SGB as per the experimental requirements. We discovered that the rats in the SAP-ALI group displayed pulmonary capillary hyperemia, plentiful inflammatory cytokine infiltration in the alveolar cavity, interstitial edema, destroyed alveolar structure, and thickened alveolar wall. In contrast with the control group, the SAP-ALI group gained a substantially higher pathological score. However, the SAP-ALI + SGB group exhibited no distinct pathological alterations, with a score much lower than that of the SAP-ALI group but higher than that of the SGB group (*P* < *0.05*, Fig. 1A). As evidenced by the dry and wet method, the lung water content was obviously heightened in the SAP-ALI group as opposed to the sham group. Lung edema was alleviated in the SAP-ALI + SGB group, less than the SAP-ALI group but more than the SGB group (*P* < *0.05*, Fig. 1B). Arterial blood gas analysis disclosed that by contrast to the sham group, the SAP-ALI group witnessed a notable drop in the levels of PH and PaO_2_ and a rise in the level of PaCO_2_. As compared with the SAP-ALI group, the SAP-ALI + SGB group experienced a rise in PH and PaO_2_ and a decline in PaCO_2_. When compared to the SGB group, the SAP-ALI + SGB group went through a decrease in PH and PaO_2_ and an increase in PaCO_2_ (*P* < *0.05*, Fig. 1C–E). We examined serum amylase, ALT, and AST and uncovered that in contrast with the sham group, there was a remarkable uplift in serum amylase, ALT, and AST in the SAP-ALI group. The levels of these three indicators in the SAP-ALI + SGB group were evidently fewer than in the SAP-ALI group but more than in the SGB group (*P* < *0.05*, Fig. 1F–H). These findings denoted that SGB could conspicuously abate ALI induced by SAP in rats.

### SGB mitigated SAP-elicited inflammation

**Fig. 2:**
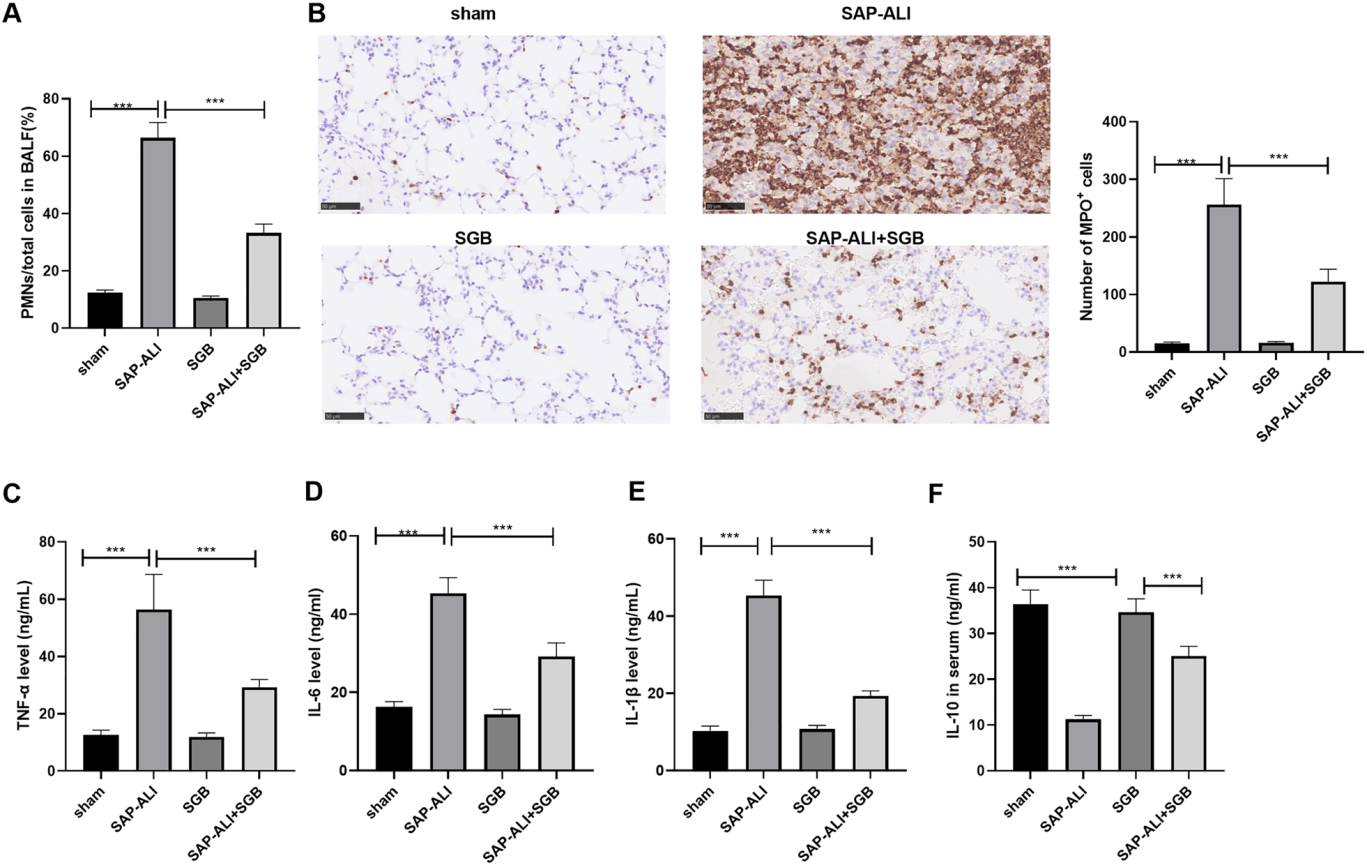
SGB alleviated inflammation induced by SAP. The abdominal aortic blood of the SAP-ALI model rats was harvested. After the animals were narcotized, a bronchoscope was utilized for lavage with normal saline. Purified PMNs were derived from the obtained BALF. **A** The PMN/total cell proportion in BALF was measured. **B** Immunohistochemistry examined neutrophils marked by MPO. **C**–**F** ELISA kits were adopted to check the profiles of inflammatory cytokines (TNF-α, IL-6, IL-1β, IL-10) in the rat lung tissues. ^***^P < *0.001* (vs. the sham group); ^***^P < *0.001* (vs. the SAP-ALI group). *N* = 5

We dug deeper into rat-associated inflammation induced by SAP. With the rat BALF harvested, we discovered that the PMN/total cell proportion was notably heightened in the SAP-ALI group against the sham group, and the proportion was much lower in the SAP-ALI + SGB group than the SAP-ALI group (*P* < *0.05*, Fig. 2A). Immunohistochemistry examined neutrophils marked by MPO, indicating that in contrast with the sham group, the SAP-ALI group witnessed a substantial increase in positive MPO cells, but SGB attenuated the number of these cells (Fig. 2B). ELISA evaluated inflammatory cytokines, displaying that by contrast to the sham group, there was a rise in pro-inflammatory factors TNF-α, IL-6, and IL-1β and a decline in the anti-inflammatory factor IL-10 in the SAP-ALI group. As opposed to the SAP-ALI group, the SAP-ALI + SGB group distinctly hampered pro-inflammatory responses induced by SAP-ALI and upregulated IL-10. When compared to the SGB group, the levels of TNF-α, IL-6, and IL-1β were heightened, and the level of IL-10 was lowered in the SAP-ALI + SGB group (*P* < *0.05*, Fig. 2C–F). Given these phenomena, SGB could dampen inflammatory factor release induced by SAP.

### SGB suppressed miR-155-5p and upregulated SOCS5

**Fig. 3:**
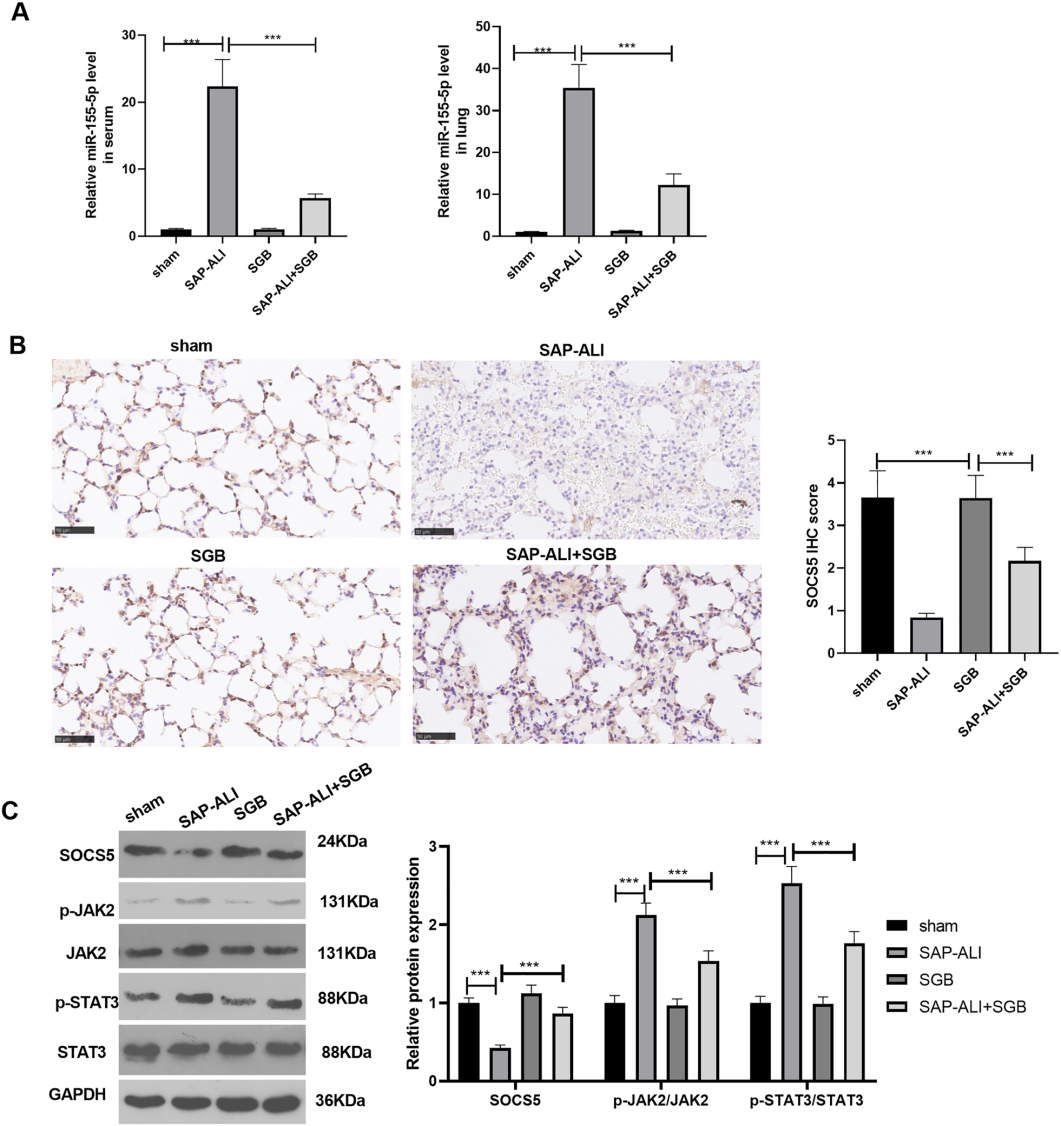
SGB dampened miR-155-5p and upregulated SOCS5. **A** RT-PCR gauged miR-155-5p expression in the rat plasma and lung tissues. **B** Immunohistochemistry confirmed SOCS5’s level. **C** Western blot determined the profile of the SOCS5/JAK2/STAT3 pathway in the rat lung tissues. ^***^P < *0.001* (vs. the sham group); ^***^P < *0.001* (vs. the SAP-ALI group). *N* = 5

qRT-PCR determined miR-155-5p’s level in the plasma and lung tissues. As a result, miR-155-5p’s level was dramatically augmented in the plasma and lung tissues of the SAP-ALI rats, whereas SGB failed to alter its level (in contrast with the sham group). By contrast to the SAP-ALI group, SGB gave rise to a distinct drop in miR-155-5p’s level (Fig. 3A). Then, immunohistochemistry and Western blot confirmed the levels of SOCS5 and JAK2/STAT3 in the lung tissues. As opposed to the sham group, SAP-ALI vigorously restricted SOCS5’s level and boosted JAK2 and STAT3 phosphorylation, whereas SGB dramatically augmented SOCS5 and suppressed JAK2 and STAT3 phosphorylation (Fig. 3B, C). In light of these findings, SGB could repress miR-155-5p expression and JAK2/STAT3 pathway activation and upregulate SOCS5.

### miR-155-5p targeted SOCS5

**Fig. 4:**
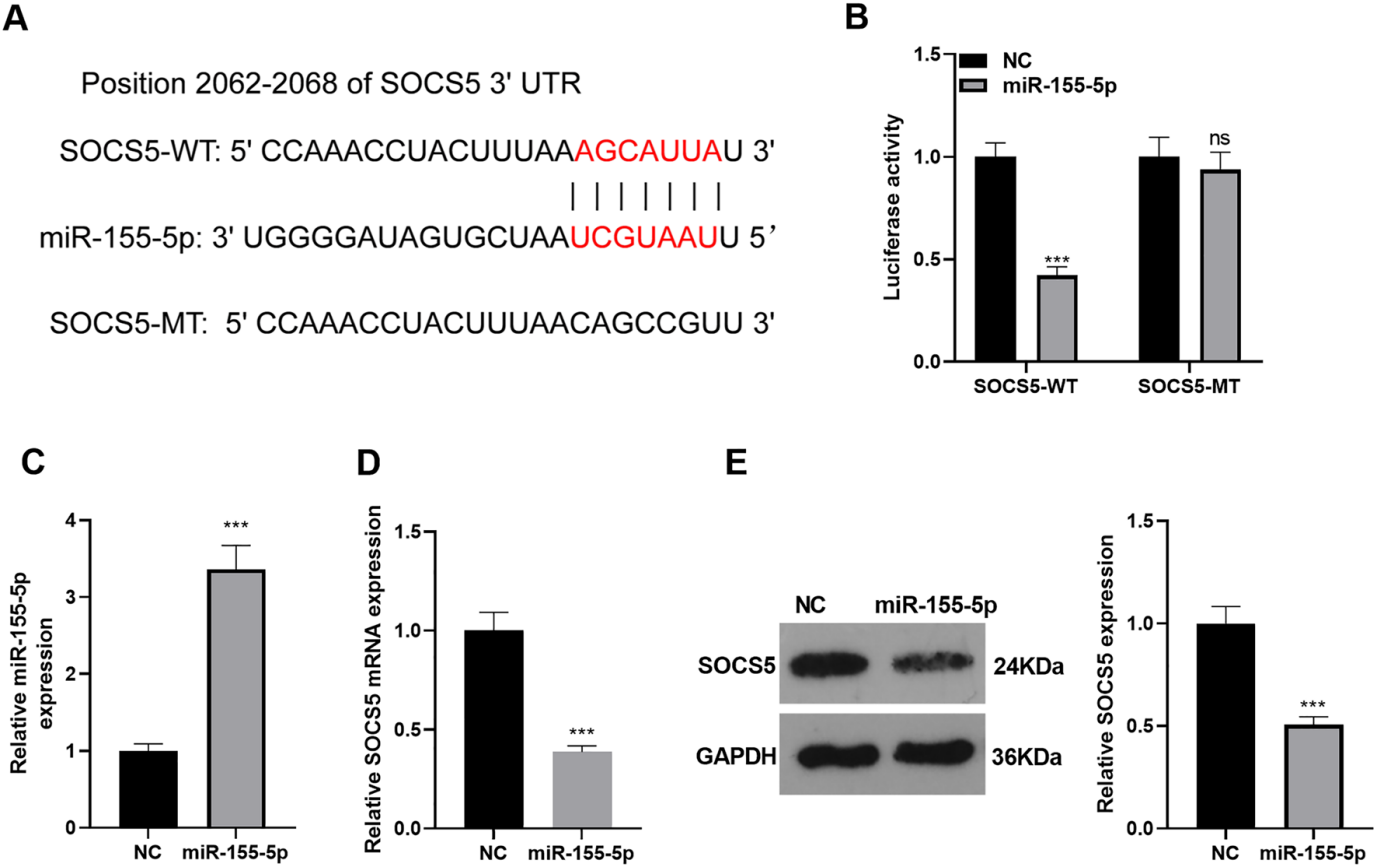
miR-155-5p targeted SOCS5. **A** Starbase (http://starbase.sysu.edu.cn/) searched the base binding diagram of miR-155-5p and SOCS5. **B** Dual luciferase activity assay confirmed SOCS5-WT and SOCS5-MT expressions following miR-155-5p overexpression. **C** A miR-155-5p overexpression model was engineered in the MLE-12 cell line. qRT-PCR ascertained miR-155-5p expression. **D** qRT-PCR figured out SOCS5 mRNA expression. **E** Western blot verified SOCS5 protein expression. *NS P* > 0.05, ***P < *0.001* (vs. the NC group). *N* = 3

Through the biological information website (http://starbase.sysu.edu.cn/), we confirmed that SOCS5 was an essential downstream target of miR-155-5p (*P* < *0.05*, Fig. 4A). Dual luciferase activity assay disclosed that miR-155-5p vigorously hindered the dual luciferase activity of the SOCS5-WT group but exerted little inhibitory impact on that of SOCS5-MT (*P* < *0.05*, Fig. 4B). miR-155-5p mimics were transfected into MLE-12 cells dealt with LPS (*P* < *0.05*, Fig. 4C). RT-PCR and Western blot displayed that miR-155-5p mimic transfection considerably restrained the protein profile of SOCS5 (*P* < *0.05*, Fig. 4D, E). These discoveries denoted that miR-155-5p targeted SOCS5 and repressed its expression.

### miR-155-5p modulated SOCS5/JAK2/STAT3 to boost MLE-12 cell damage mediated by LPS

**Fig. 5:**
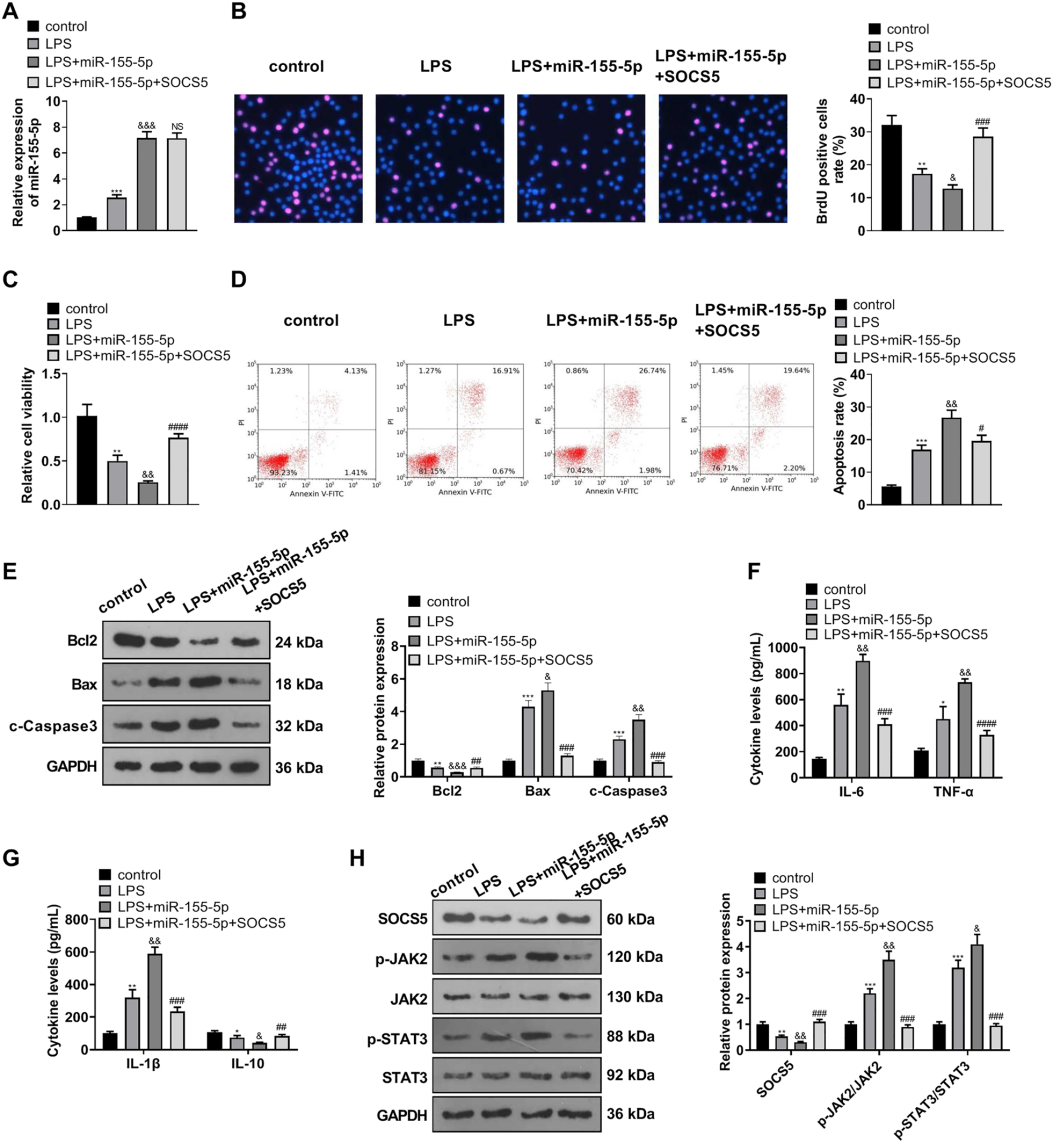
miR-155-5p modulated SOCS5/JAK2/STAT3 to step up MLE-12 cell injury mediated by LPS. MLE-12 cells were transfected along with miR-155-5p mimics and then with miR-155-5p mimics plus SOCS5 overexpression plasmid. LPS (1 μg/mL) was taken to treat the cells for 24 h. **A** qRT-PCR confirmed miR-155-5p expression in MLE-12 cells. **B**, **C** BrdU and CCK8 examined MLE-12 cell proliferation and viability. **D** Flow cytometry tracked MLE-12 apoptosis. **E** Western blot determined the profiles of apoptosis-concerned proteins in MLE-12 cells. **F**, **G** ELISA checked the levels of TNF-α, IL-6, IL-1β, and IL-10 in MLE-12 cells. **H** Western blot figured out the profile of the SOCS5/JAK2/STAT3 pathway in MLE-12 cells. *P < 0.05, **P < 0.01, ***P < *0.001* (vs. the control group); *&P* < *0.05, &&P* < *0.01, &&& P* < *0.001* (vs. the LPS group); *NS P* > *0.05, #P* < *0.05, ##P* < *0.01, ###P* < *0.001, ####P* < *0.0001* (vs. the LPS + miR-155-5p group). *N* = 3

We transfected MLE-12 cells along with miR-155-5p mimics and miR-155-5p mimics + SOCS5 overexpression plasmid. Then the cells were dealt with LPS (1 μg/mL) for 24 h. qRT-PCR unveiled that miR-155-5p expression in the LPS group was much higher than in the control group, and it was further heightened by miR-155-5p overexpression. Nevertheless, no distinct differences were discovered in miR-155-5p expression in MLE-12 cells between the LPS + miR-155-5p group and the LPS + miR-155-5p + SOCS5 group (Fig. 5A). As unraveled by BrdU and CCK8, in contrast with the control group, LPS weakened MLE-12 cell proliferation and viability. By contrast to the LPS group, miR-155-5p overexpression gave rise to a reduction in MLE-12 cell viability and proliferation, which were strengthened by SOCS5 overexpression (in contrast with the LPS + miR-155-5p group) (Fig. 5B, C). As evidenced by flow cytometry, as compared with the control group, LPS contributed to a rise in MLE-12 cells’ apoptosis. Their apoptosis was evidently facilitated by miR-155-5p overexpression (in contrast with the LPS group, Fig. 5D) and vigorously hampered by SOCS5 overexpression (by contrast to LPS + miR-155-5p, Fig. 5D). Western blot confirmed the profiles of apoptosis-concerned proteins, signifying that by contrast to the control group, LPS culminated in a rise in Bax and c-Caspase3 expressions and a decline in Bcl2 expression in MLE-12 cells, while miR-155-5p overexpression boosted the increase of Bax and c-Caspase3 expressions and the reduction of Bcl2 expression (compared to the LPS group). As opposed to LPS + miR-155-5p, SOCS5 overexpression cramped Bax and c-Caspase3 expressions and augmented Bcl2 expression (Fig. 5E). ELISA reflected that in contrast with the control group, LPS uplifted the profiles of TNF-α, IL-6, and IL-1β and attenuated the profile of IL-10 in MLE-12 cells. miR-155-5p overexpression bolstered TNF-α, IL-6, and IL-1β expressions and lowered IL-10 expression in MLE-12 cells dealt with LPS (by contrast to the LPS group). As compared with the LPS + miR-155-5p group, SOCS5 overexpression resulted in the opposite situation (Fig. 5F, G). Western blot determined the profile of SOCS5/JAK2/STAT3 in MLE-12 cells. As a result, in contrast with the control group, LPS suppressed SOCS5 expression and strengthened JAK2 and STAT3 phosphorylation, whereas miR-155-5p overexpression lowered SOCS5 expression and uplifted JAK2 and STAT3 phosphorylation levels (by contrast to the LPS group). When compared to the LPS + miR-155-5p group, SOCS5 overexpression substantially enhanced SOCS5 expression and weakened JAK2 and STAT3 phosphorylation (Fig. 5H). These findings revealed that miR-155-5p modulated SOCS5/JAK2/STAT3 to bolster MLE-12 cell damage mediated by LPS, and SOCS5 overexpression weakened the promoting influence of miR-155-5p overexpression on MLE-12 cell injury.

### STAT3 inhibition weakened the damaging function of miR-155-5p overexpression in MLE-12 cells

**Fig. 6:**
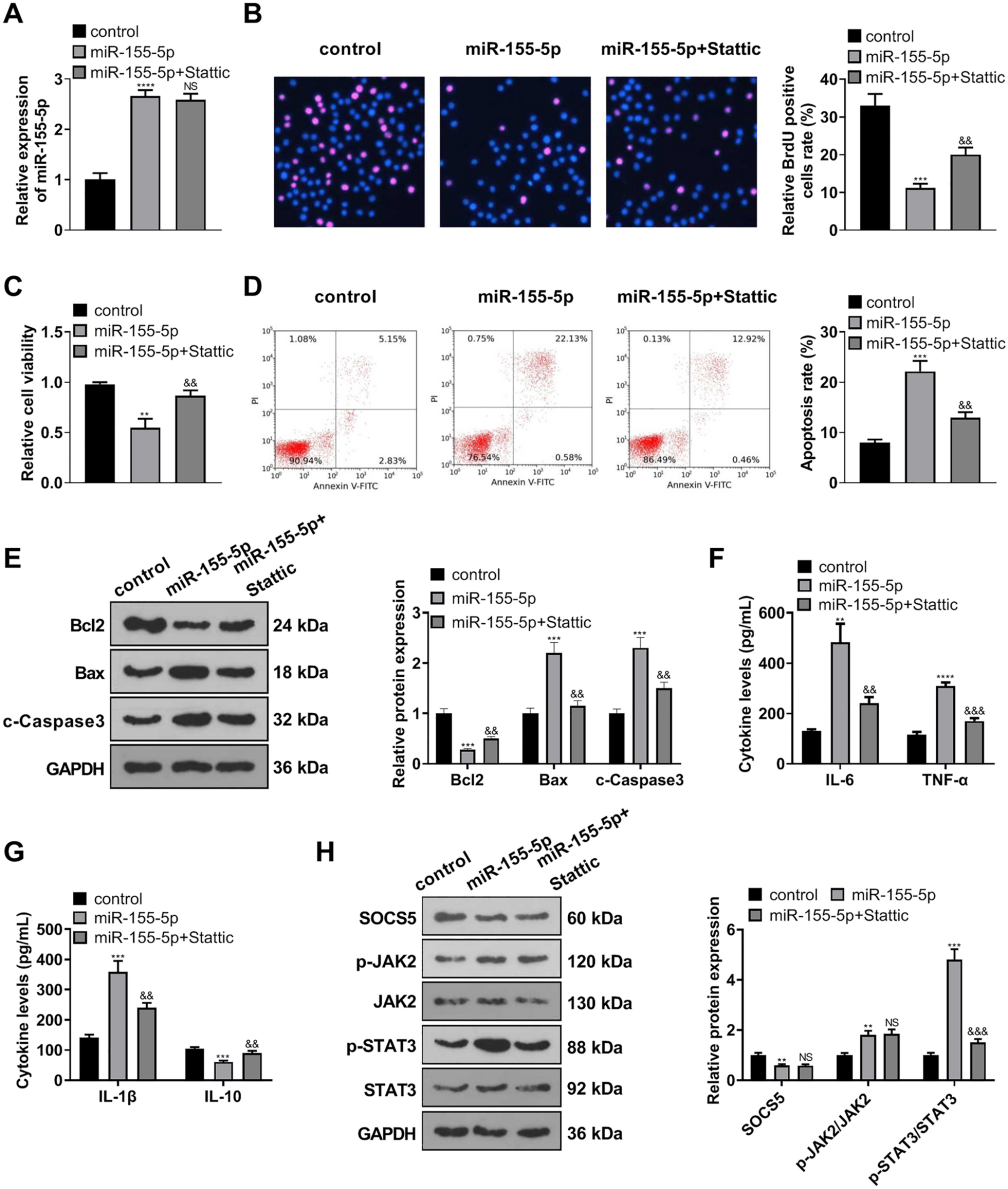
STAT3 inhibition weakened the damaging function of miR-155-5p overexpression in MLE-12 cells. MLE-12 cells were transfected along with miR-155-5p mimics, and then the STAT3 inhibitor Stattic was administered. **A** qRT-PCR confirmed miR-155-5p expression in MLE-12 cells. **B**, **C** BrdU and CCK8 measured MLE-12 viability and proliferation. **D** Flow cytometry tracked MLE-12 apoptosis. **E** Western blot determined the profiles of apoptosis-associated proteins in MLE-12 cells. **F**, **G** ELISA ascertained the profiles of TNF-α, IL-6, IL-1β, and IL-10 in MLE-12 cells. **H** Western blot gauged the profile of SOCS5/JAK2/STAT3 in MLE-12 cells. **P < 0.01, ***P < 0.001, ****P < *0.0001* (vs. the control group); *NS P* > *0.05, &&P* < *0.01, &&&P* < *0.001* (vs. the miR-155-5p group). *N* = 3

miR-155-5p mimics were transfected into MLE-12 cells, followed by the administration of the STAT3 inhibitor Stattic. qRT-PCR displayed that no conspicuous differences in miR-155-5p expression were discovered in MLE-12 cells between the miR-155-5p group and the miR-155-5p + Stattic group (Fig. 6A). As supported by BrdU and CCK8, by contrast to the control group, miR-155-5p overexpression abated MLE-12 cell viability and proliferation, whereas Stattic enhanced the viability and proliferation of MLE-12 cells transfected along with miR-155-5p mimics (in contrast with the miR-155-5p group) (Fig. 6B, C). Flow cytometry disclosed that by contrast to the control group, miR-155-5p overexpression gave rise to an increase in MLE-12 cell apoptosis, which was vigorously frustrated by Stattic (against the miR-155-5p group, Fig. 6D). Western blot ascertained the profiles of apoptosis-concerned proteins and uncovered that as compared with the control group, miR-155-5p overexpression bolstered Bax and c-Caspase3 expressions and lowered Bcl2 expression, but Stattic inverted the phenomena (against the miR-155-5p group) (Fig. 6E). ELISA reflected that in contrast with the control group, miR-155-5p overexpression heightened TNF-α, IL-6, and IL-1β expressions and repressed IL-10 expression in MLE-12 cells, but such a situation was reversed by Stattic (against the miR-155-5p group) (Fig. 6F, G). Western blot examined the profile of SOCS5/JAK2/STAT3 in MLE-12 cells. It transpired that by contrast to the control group, miR-155-5p overexpression drove up JAK2 and STAT3 phosphorylation levels. However, STAT3 phosphorylation was impaired, while SOCS5 expression and JAK2 phosphorylation remained basically the same in the miR-155-5p + Stattic group vis-a-vis the miR-155-5p group (Fig. 6H). All these findings confirmed that STAT3 inhibition weakened the damaging function of miR-155-5p overexpression in MLE-12 cells.

## Discussion

As the economy develops rapidly and living standards improve, the intake of high-fat and high-protein food and drinks is also on the rise, which adds to the burden of the digestive system. Gastroenterology patients are usually hospitalized because of acute pancreatitis (AP). Reportedly, 33.74 of every 100,000 people would develop AP every year [20], and the incidence rate continues to increase. Around 10% of AP patients will evolve to severe acute pancreatitis (SAP) [21], which is often accompanied by severe organ dysfunctions, among them acute lung injury (ALI) a common one. SAP-ALI occurrence and progression may pertain to trypsin activation, inflammation, oxidative stress, regulatory functions of microRNAs in downstream pathways, and other mechanisms [22]. Stellate ganglion block, a prevailing treatment strategy, regulates abnormalities in the neuro-endocrine system through local blocking without incurring central nervous system impairment while upholding the integrity of the surrounding nerve. Through experiments, we discovered that SGB vigorously dampened rat lung damage elicited by SAP, which demonstrated that SGB might be utilized for SAP-ALI treatment.

Stellate ganglion block (SGB) is known as a century-old technology that blocks the sympathetic nerve chains in the cervical spine, upper chest and other places for diagnosis and treatment. SGB has been substantiated to ameliorate trigeminal neuropathy and paresthesia following dental surgery [23], post-traumatic stress disorder syndrome [24], sleep disorders in patients with breast cancer, etc [25]. Moreover, it also exerts a good therapeutic function in lung damage resulting from multiple factors. SGB vigorously suppresses the sepsis-elicited release of inflammatory factors IL-6 and TNF-α, alleviating ALI in rats [26]. In the rabbit ALI model elicited by hydrochloric acid, SGB could attenuate stress responses, modulate homeostasis in the autonomic nervous system, substantially abate the profiles of pro-inflammatory factors IL-6 and TNF-α, and upregulates IL-10, thus enhancing the functions of lungs affected by ALI [4]. Unfortunately, it is still poorly understood whether SGB can function in SAP-ALI and how it exactly works. Given the above findings, we can know that the function of SGB to ameliorate pulmonary functions may be correlated with anti-inflammatory responses. Luckily, our experiments demonstrated that SGB vigorously repressed pathological lesions in the lungs of the SAP-ALI rats, efficaciously enhanced their ventilation functions, distinctly lowered the levels of serum amylase, ALT, and AST and the PMN/total cell proportion, and also conspicuously weakened pro-inflammatory cytokine release.

miRNAs play a pivotal part in gene expression, mediating the occurrence and progression of umpteen diseases. Of note, many miRNAs now are extensively adopted as biomarkers for diagnosis, treatment, and prognosis. For instance, miR-216a-5p, miR-216b-5p, miR-217-5p, and miR-375-3p, whose sensitivity is higher than serum amylase and lipase, can perfectly serve as biomarkers for AP [27]. miR-22-3p, miR-1260b, miR-762, miR-23b, and miR-23a are all considerably upregulated in the context of SAP-ALI [28]. miR-155 targets SOCS1 to strengthen inflammation mediated by Th17 in AP, while miR-155 inhibition vigorously impedes inflammation and ameliorates pancreatic pathology [29]. miR-155 is also a pro-inflammatory regulator inextricably associated with exacerbated AP, but miR-155 inhibition can mitigate ALI in cerulein-elicited AP mice [30]. miR-155-5p targets DUSP14 and initiates the NF-κB and MAPKs signaling pathways to boost OGD/R-elicited SH-SY5Y cell apoptosis and inflammation [31]. lncRNA CTBP1-AS2 targets miR-155-5p and upregulates FOXO1 to abate HG-elicited proliferation, oxidative stress, ECM accumulation, and inflammation in human glomerular mesangial cells [32]. lncRNA XIST targets miR-155-5p and upregulates WWC1 to dampen inflammatory cytokine generation and cell apoptosis, hence attenuating acute kidney injury induced by sepsis [33]. Here, we discovered that miR-155-5p’s expression was greatly uplifted in the plasma and lung tissues of the SAP-ALI rats, but SGB notably drove down its expression. In vitro, miR-155-5p overexpression remarkably facilitated MLE-12 cells’ apoptosis and inflammation and weakened their proliferation and viability. miR-155-5p overexpression exacerbated MLE-12 cell injury mediated by LPS. All these findings confirmed that miR-155-5p displayed pro-apoptotic and pro-inflammatory functions in the context of SAP-ALI.

SOCS5 is characterized as a specific inhibitor of IL-4 signaling [34]. During COPD progression, miR-132 overexpression targets and hampers SOCS5 to bolster EGFR protein expression and inflammatory cytokine generation in human monocyte-like cells (THP-1) [35]. The JAK2/STAT3 signaling pathway exerts an outstanding function in lung damage. For instance, miR-216a overexpression suppresses JAK2/STAT3 and NF-κB pathway activation to mitigate ALI induced by LPS [36]. SOCS3 overexpression represses JAK2/STAT3 pathway activation and inflammatory factor production to facilitate the repair of rat lung damage elicited by SAP [37]. Leonurine down-regulates miR-18a-5p and enhances SOCS5 expression to curb JAK2/STAT3 signaling pathway activation, impeding CML cells’ proliferation, migration, and colony formation, boosting their apoptosis, and thus exerting a prominent anti-leukemia function in chronic myeloid leukemia [38]. Notwithstanding, we are still in the dark about the mechanism of the SOCS5/JAK2/STAT3 pathway in lung damage elicited by pancreatitis. Here, we uncovered a notable drop in SOCS5 expression and immunohistochemical scores and a rise in JAK2 and STAT3 phosphorylation in SAP-ALI rat lung tissues. Nevertheless, SGB culminated in a distinct uplift in SOCS5 expression and immunohistochemical scores and a remarkable decline in JAK2 and STAT3 phosphorylation. The bioinformatics website denoted that SOCS5 might be a downstream target of miR-155-5p, which was substantiated by dual luciferase activity assay. In vitro, miR-155-5p overexpression restrained SOCS5 expression. SOCS5 overexpression abated the promoting influence of miR-155-5p overexpression on MLE-12 cell injury mediated by LPS and attenuated JAK2 and STAT3 phosphorylation. STAT3 inhibition weakened the damaging function of miR-155-5p overexpression in MLE-12 cells. Therefore, we speculated that SGB could down-regulate miR-155-5p and enhance SOCS5 expression to curb JAK2/STAT3 pathway activation, thereby ameliorating SAP-ALI.

## Conclusion

**Fig.7:**
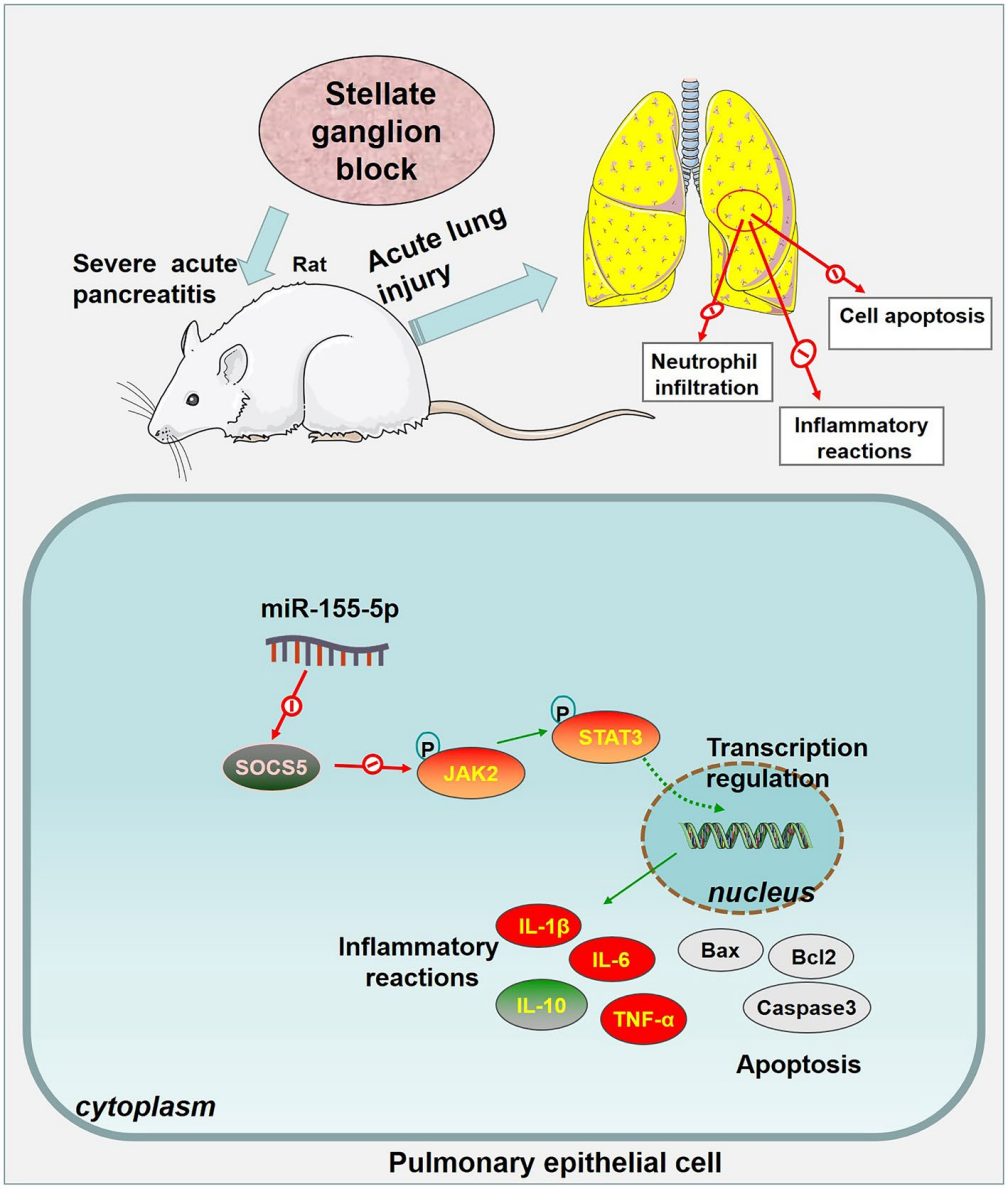
The diagram of stellate ganglion block (SGB) in ameliorating SAP-induced ALI (SAP-ALI) by regulating miR-155-5p/SOCS5/JAK2/STAT3 axis

To summarize, we have uncovered that SGB can prominently alleviate SAP-ALI through engineering an SAP-ALI rat model and treating it with SGB. SGB bolsters the profile of SOCS5, represses miR-155-5p expression, and impedes JAK2/STAT3 pathway activation. miR-155-5p induces JAK2/STAT3 pathway activation by directly targeting SOCS5 (Fig. 7). However, several limitations remain to be investigated in our future study: (1) lack of clinical trial; (2) lack of in vivo experiments on genetic modification of miR-155-5p/SOCS5 on stellate ganglion block-mediated effects. All in all, our finding provides a new reference in treating SAP-ALI by SGB.

## Data Availability

The data sets used and analyzed during the current study are available from the corresponding author on reasonable request.

## References

[1] Yadav D, Lowenfels AB (2013). The epidemiology of pancreatitis and pancreatic cancer. Gastroenterology.

[2] Elder AS, Saccone GT, Dixon DL (2012). Lung injury in acute pancreatitis: mechanisms underlying augmented secondary injury. Pancreatology.

[3] Jacobs ML, Daggett WM, Civette JM (1977). Acute pancreatitis: analysis of factors influencing survival. Ann Surg.

[4] Liu Y, Tao T, Li WZ (2017). Regulating autonomic nervous system homeostasis improves pulmonary function in rabbits with acute lung injury. BMC Pulm Med.

[5] Wang ZY, Li SY, Lai HZ (2019). Interaction between endothelin-1 and left stellate ganglion activation: a potential mechanism of malignant ventricular arrhythmia during myocardial ischemia. Oxid Med Cell Longev.

[6] Summers MR, Nevin RL (2017). Stellate ganglion block in the treatment of post-traumatic stress disorder: a review of historical and recent literature. Pain Pract.

[7] Wang WW, Shi WD, Qian H (2017). Stellate ganglion block attenuates chronic stress induced depression in rats. PLoS ONE.

[8] Zhao HY, Yang GT, Sun NN (2017). Efficacy and safety of stellate ganglion block in chronic ulcerative colitis. World J Gastroenterol.

[9] Dexheimer PJ, Cochella L (2020). MicroRNAs: from mechanism to organism. Front Cell Dev Biol.

[10] Liu P, Xia L, Zhang WL (2014). Identification of serum microRNAs as diagnostic and prognostic biomarkers for acute pancreatitis. Pancreatology.

[11] Zhao PY, Zang B, Gong XY (2020). MiR-214-3p exacerbates kidney damages and inflammation induced by hyperlipidemic pancreatitis complicated with acute renal injury. Life Sci.

[12] Zhou X, Chen J, Tao H (2020). Intranasal delivery of miR-155-5p Antagomir Alleviates acute seizures likely by inhibiting hippocampal inflammation. Neuropsychiatr Dis Treat.

[13] Lv R, Du L, Zhou F, Yuan X, Liu X, Zhang L (2020). Rosmarinic acid alleviates inflammation, apoptosis, and oxidative stress through regulating miR-155-5p in a mice model of Parkinson’s disease. ACS Chem Neurosci.

[14] Li HF, Wu YL, Tseng TL, Chao SW, Lin H, Chen HH (2020). Inhibition of miR-155 potentially protects against lipopolysaccharide-induced acute lung injury through the IRF2BP2-NFAT1 pathway. Am J Physiol Cell Physiol.

[15] Jumaa H, Wei G, Nielsen PJ (1999). Blastocyst formation is blocked in mouse embryos lacking the splicing factor SRp20. Curr Biol.

[16] Cooney RN (2002). Suppressors of cytokine signaling (SOCS): inhibitors of the JAK/STAT pathway. Shock.

[17] Fu Y, Xu Y, Chen S, Ouyang Y, Sun G (2020). MiR-151a-3p promotes postmenopausal osteoporosis by targeting SOCS5 and activating JAK2/STAT3 signaling. Rejuvenation Res.

[18] Mohamed EA, Sayed WM (2020). Implication of JAK1/STAT3/SOCS3 pathway in aging of cerebellum of male rat: histological and molecular study. Sci Rep.

[19] Piao XH, Zou YP, Sui XD (2019). Hydrostatin-SN10 ameliorates pancreatitis-induced lung injury by affecting IL-6-induced JAK2/STAT3-associated inflammation and oxidative stress. Oxid Med Cell Longev.

[20] Xiao AY, Tan ML, Wu LM (2016). Global incidence and mortality of pancreatic diseases: a systematic review, meta-analysis, and meta-regression of population-based cohort studies. Lancet Gastroenterol Hepatol.

[21] Banks PA, Bollen TL, Dervenis C (2013). Classification of acute pancreatitis–2012: revision of the Atlanta classification and definitions by international consensus. Gut.

[22] Ge P, Luo YL, Okoye CS (2020). Intestinal barrier damage, systemic inflammatory response syndrome, and acute lung injury: a troublesome trio for acute pancreatitis. Biomed Pharmacother.

[23] Sago T, Takahashi O, Ogawa M (2020). Effects of stellate ganglion block on postoperative trigeminal neuropathy after dental surgery: a propensity score matching analysis. Sci Rep.

[24] Hanling SR, Hickey A, Lesnik I (2016). Stellate ganglion block for the treatment of posttraumatic stress disorder: a randomized, double-blind controlled trial. Reg Anesth Pain Med.

[25] Haest K, Kumar A, Van Calster B (2012). Stellate ganglion block for the management of hot flashes and sleep disturbances in breast cancer survivors: an uncontrolled experimental study with 24 weeks of follow-up. Ann Oncol.

[26] Chen Y, Guo L, Lang HL (2018). Effect of a stellate ganglion block on acute lung injury in septic rats. Inflammation.

[27] Erdos Z, Barnum JE, Wang E (2020). Evaluation of the relative performance of pancreas-specific MicroRNAs in rat plasma as biomarkers of pancreas injury. Toxicol Sci.

[28] Lu XG, Kang X, Zhan LB (2017). Circulating miRNAs as biomarkers for severe acute pancreatitis associated with acute lung injury. World J Gastroenterol.

[29] Wang DY, Tang MC, Zong PF (2018). MiRNA-155 regulates the Th17/Treg ratio by targeting SOCS1 in severe acute pancreatitis. Front Physiol.

[30] Wan JH, Yang XY, Ren YP (2019). Inhibition of miR-155 reduces impaired autophagy and improves prognosis in an experimental pancreatitis mouse model. Cell Death Dis.

[31] Shi Y, Li K, Xu K, Liu QH (2020). MiR-155-5p accelerates cerebral ischemia-reperfusion injury via targeting DUSP14 by regulating NF-κB and MAPKs signaling pathways. Eur Rev Med Pharmacol Sci.

[32] Wang G, Wu B, Zhang B, Wang K, Wang H (2020). LncRNA CTBP1-AS2 alleviates high glucose-induced oxidative stress, ECM accumulation, and inflammation in diabetic nephropathy via miR-155-5p/FOXO1 axis. Biochem Biophys Res Commun.

[33] Wang L, Cao QM (2021). Long non-coding RNA XIST alleviates sepsis-induced acute kidney injury through inhibiting inflammation and cell apoptosis via regulating miR-155–5p/WWC1 axis. Kaohsiung J Med Sci.

[34] Kubo M, Ozaki A, Tanaka S, Okamoto M, Fukushima A (2006). Role of suppressor of cytokine signaling in ocular allergy. Curr Opin Allergy Clin Immunol.

[35] Diao X, Zhou J, Wang S, Ma X (2018). Upregulation of miR-132 contributes to the pathophysiology of COPD via targeting SOCS5. Exp Mol Pathol.

[36] Kong F, Sun Y, Song W, Zhou Y, Zhu S (2020). MiR-216a alleviates LPS-induced acute lung injury via regulating JAK2/STAT3 and NF-κB signaling. Hum Cell.

[37] Qin MZ, Qin MB, Liang ZH, Tang GD (2019). Effect of SOCS3 on lung injury in rats with severe acute pancreatitis through regulating JAK2/STAT3 signaling pathway. Eur Rev Med Pharmacol Sci.

[38] Liu HM, Guo CL, Zhang YF, Chen JF, Liang ZP, Yang LH, Ma YP (2021). Leonurine-Repressed miR-18a-5p/SOCS5/JAK2/STAT3 Axis Activity Disrupts CML malignancy. Front Pharmacol.

